# Combined application of block and modulation factors to reduce the volume of the low dose area in helical tomotherapy plans for lung cancer

**DOI:** 10.1002/acm2.70372

**Published:** 2025-12-10

**Authors:** Sijin Zhu, Tianwen Zhang, Yutao Zhao, Jinli Peng, Jingyan Gao, Yunyan Yang, Yan Xu, Jiawen Yan, Ya Li

**Affiliations:** ^1^ Department of Radiation Oncology Yunnan Cancer Hospital The Third Affiliated Hospital of Kunming Medical University Peking University Cancer Hospital Yunnan Kunming Yunnan China

**Keywords:** block, helical tomotherapy, lung cancer, modulation factors, pneumonitis

## Abstract

**Background:**

The helical tomotherapy (HT) system can expose more normal lung tissue to low radiation doses, resulting in extensive low‐dose distribution in both lungs which may induce radiation pneumonitis (RP).

**Purpose:**

This study aims to optimize dosimetric parameters and identify a clinically feasible treatment plan by analyzing the impact of different block settings and modulation factor (MF) combinations on HT plans for lung cancer to reduce the low‐dose exposure volumes in normal lung tissue.

**Methods:**

We retrospectively reviewed 14 lung cancer patients who received radiotherapy. These cases were optimized using different modulation factors (MFs: 3, 4, 5) and block techniques (Unblocked, Directional, Complete). The impact of MF and block combinations on reducing low‐dose bath (e.g., V5, V10) in lung tissue was evaluated by analyzing dose distribution maps, dose–volume histograms (DVH), homogeneity index (HI), conformity index (CI), and treatment time for each optimized HT plan combination.

**Results:**

Block settings and higher MF exerted minimal influence on the average dose to the target volume. With stricter block constraints and higher MF, HI increased (range: 17.39%–27.54%) and CI decreased (range: 2.76%–17.43%); although both indices showed slight deterioration, they remained within acceptable clinical limits. Treatment time increased substantially (range: 15.77%–131.58%). The block technique significantly reduced V5 in the bilateral lungs and the contralateral (healthy) lung, with less impact on the ipsilateral (affected) lung. The combination of block and high MF effectively reduced V5.

**Conclusions:**

To balance target volume coverage (high dose), dose distribution uniformity, and treatment duration while reducing low‐dose irradiation to normal lung tissue, we recommend implementing Directional block with an MF range of 3–4 to optimize the HT plan for patients with unilateral lung cancer.

## 1 INTRODUCTION

Lung cancer has one of the highest global incidence rates among malignant tumors and is the leading cause of cancer‐related mortality.^[^ ^1^ ^]^ Radiotherapy plays a crucial role in the comprehensive management of lung cancer. Radiation pneumonitis (RP), a dose‐limiting toxicity of thoracic radiotherapy, adversely affects the therapeutic index. As a radiosensitive organ, the lung's tolerance limits radiation dose delivery to tumors. Multiple studies demonstrate a strong linear correlation between RP incidence and low‐dose irradiated lung volumes.^[^ ^2^, ^3^, ^4^, ^5^, ^6^ ^]^ In addition, low‐dose irradiated lung volumes such as V5 and V10 have been reported to be important predictive factors for RP,^[^ ^7^, ^8^, ^9^ ^]^ with V5 being a particularly significant predictor.^[^ ^10^, ^11^ ^]^ The National Comprehensive Cancer Network (NCCN Guidelines Version 2.2024) recommends the following normal tissue constraints for conventionally fractionated lung radiotherapy: V5 ≤ 65% and mean lung dose (MLD) ≤ 20 Gy. Consequently, V5 ≤ 65% is widely adopted in clinical practice as a key dosimetric constraint for normal lung tissue.^[^ ^8^ ^]^

Helical tomotherapy (HT), an intensity‐modulated radiation therapy technique utilizing helical CT scanning, achieves both excellent dose conformality and high homogeneity.^[^ ^12^ ^]^ Compared with conventional IMRT, HT demonstrates superior dose homogeneity.^[^ ^13^ ^]^ However, its 360° rotational delivery increases low‐dose exposure to distal normal lung tissue, generating an extended low‐dose bath effect in organs distant from targets. As HT technology proliferates, multi‐directional beam crossing has been associated with extensive bilateral lung dose distributions and consequent radiation pneumonitis (RP) risk.^[^ ^14^ ^]^

In this study, we aimed to optimize HT plans for lung cancer by adjusting spatial blocking techniques and modulation factor (MF) settings. This approach seeks to provide a clinically feasible treatment strategy for reducing low‐dose irradiated lung volumes (e.g., V5/V10), which may mitigate RP risk.

## 2 METHODS

### 2.1 Patients and target delineation

We retrospectively reviewed 14 lung cancer patients who received radiotherapy at our Cancer Center Hospital from November 2020 to November 2021. The local tumor TNM stage of the patients was as follows: T (2‐4) N (2‐4) M (0‐1), IIA‐IVB; Which lung lobes were the tumors located: left upper lobe = 5, left pulmonary hilum = 6, left lower lobe = 3; patients underwent supine CT simulation (slice thickness: 3–5 mm) with thermoplastic mask immobilization covering the neck and chest. Clinical target volumes (CTV) and organs at risk (OARs) were contoured by a radiation oncologist (attending physician or higher) following ICRU Report 83 guidelines. Planning target volumes (PTV) were generated by expanding CTV by 6–8 mm. The volume of PTV (68.21–137.93) cm^3^ (average 99.896 ± 20.709) cm^3^. How central were the target volumes: 0–4.76 cm, and central thoracic target volumes abutting the heart and esophagus were observed in 50% of patients (distance to center is 0 cm), The impact of PTV characteristics on the study is shown in Table S1.

### 2.2 Treatment planning

This study utilized a field width of 2.5 cm and a pitch of 0.287. The modulation factor (MF) was set to 3, 4, and 5. The block protection region covered the lateral half of the lung on the healthy side (contralateral lung) and was set to Unblocked, Directional, or Complete mode.

**FIGURE 1:**
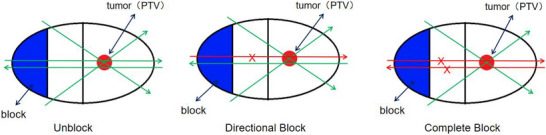
Schematic diagram of the three block settings.

In Figure 1, the green arrow indicates pathways where the beam is permitted, and the red arrow indicates pathways where the beam is prohibited. The behavior of the beam relative to the block protection region was defined as follows for each mode: Unblocked: The beam is unrestricted; it can both enter the region to reach the tumor and exit the region after passing through the tumor. Directional: The beam cannot enter the region to reach the tumor (i.e., cannot pass through the region before reaching the tumor), but it is allowed to exit through the region after passing through the tumor. Complete: The beam is prohibited from both entering the region to reach the tumor and exiting through the region after passing through the tumor.

For each of the 14 enrolled radiotherapy cases, treatment plans were optimized using all combinations of modulation factor (3, 4, or 5) and block protection mode (Unblocked, Directional, or Complete). The remaining optimization parameters were held constant across all plans. An experienced medical physicist generated nine distinct plans per case, resulting in a total of 126 plans (14 cases × 9 plans/case). All plans met the following PTV dose constraints: V_66_ Gy < 5%, V_60_ Gy > 95%, V_57_ Gy > 90%. Organ‐at‐risk dose constraints were established based on the fourth edition of “Radiation Oncology” and international guidelines for lung cancer radiotherapy. The specific quantitative constraints applied are detailed in Tables 5 and 6.

### 2.3 Evaluation

Dose parameter calculation uniformity is represented by the uniformity index (HI), HI = (D2%—D98%)/D50%, where D2%, D98%, D50% represent the doses corresponding to 2%, 98%, 50% of the PTV volume, respectively, the smaller the HI value, the better the dose uniformity; conformity is represented by the conformity index (CI), CI = (Vt, ref/Vt) · (Vt, ref/Vref), where Vt, ref is the target volume encompassed by the reference isodose line, Vt is the target volume, Vref is the volume of all areas encompassed by the reference isodose line. The closer the CI value is to 1, the better the dose conformity of the target area.

### 2.4 Statistics

Statistical analyses were performed using IBM SPSS Statistics (Version 27) to compare the differences in data between different groups using paired t‐tests. The results of the data are recorded as mean ± standard deviation. *p *< 0.05, the difference is considered statistically significant.

## 3 RESULTS

### 3.1 Dose distribution and dose–volume histogram (DVH) in one patient

**FIGURE 2:**
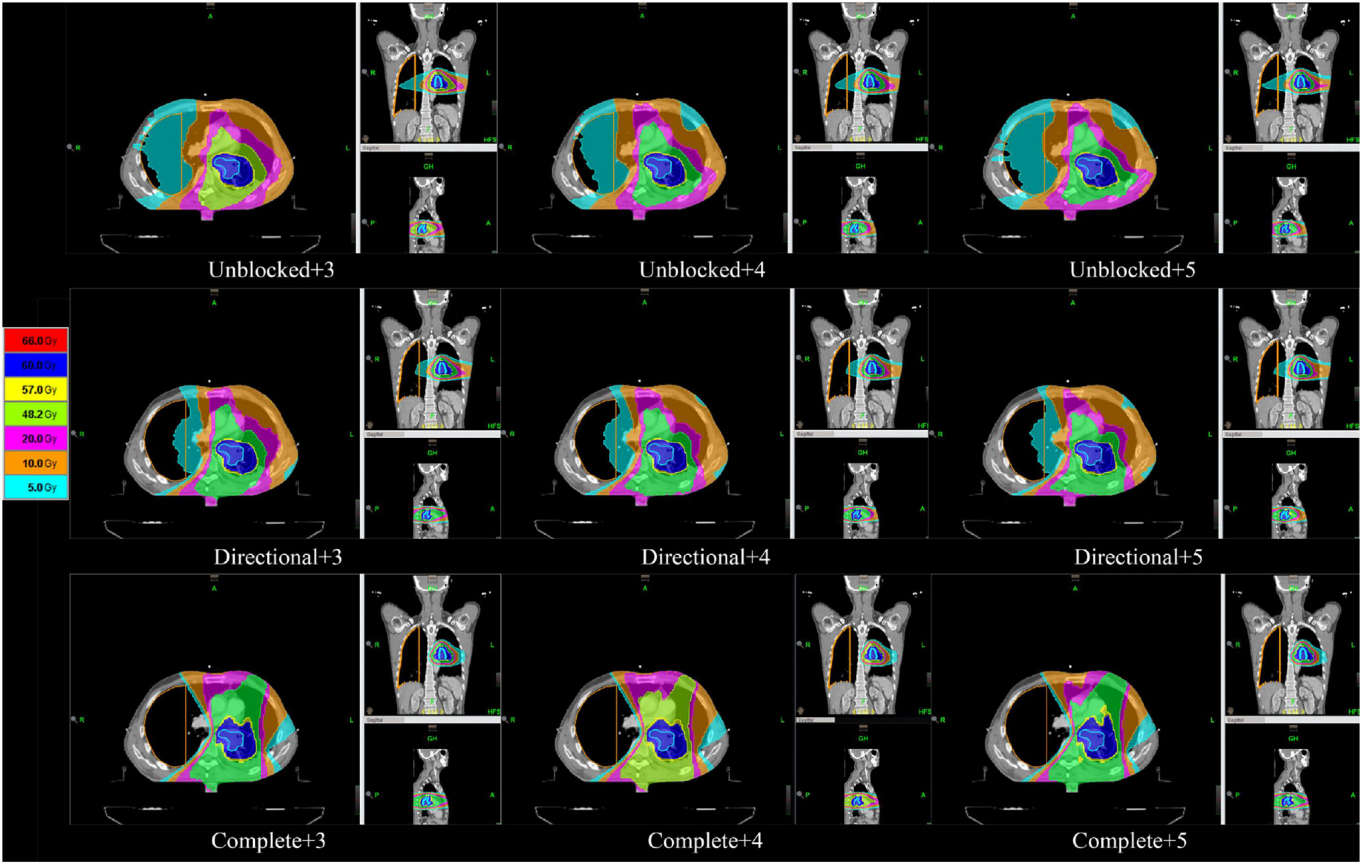
Dose distribution in the target area for various optimized combinations in a patient. Note:The light‐blue regions represent volumes receiving ≥5 Gy (V5Gy). Comparative analysis demonstrates a significant reduction in V5 (light‐blue areas) with directional block + 3/4‐field technique versus the unblocked + 3‐field approach.

**FIGURE 3:**
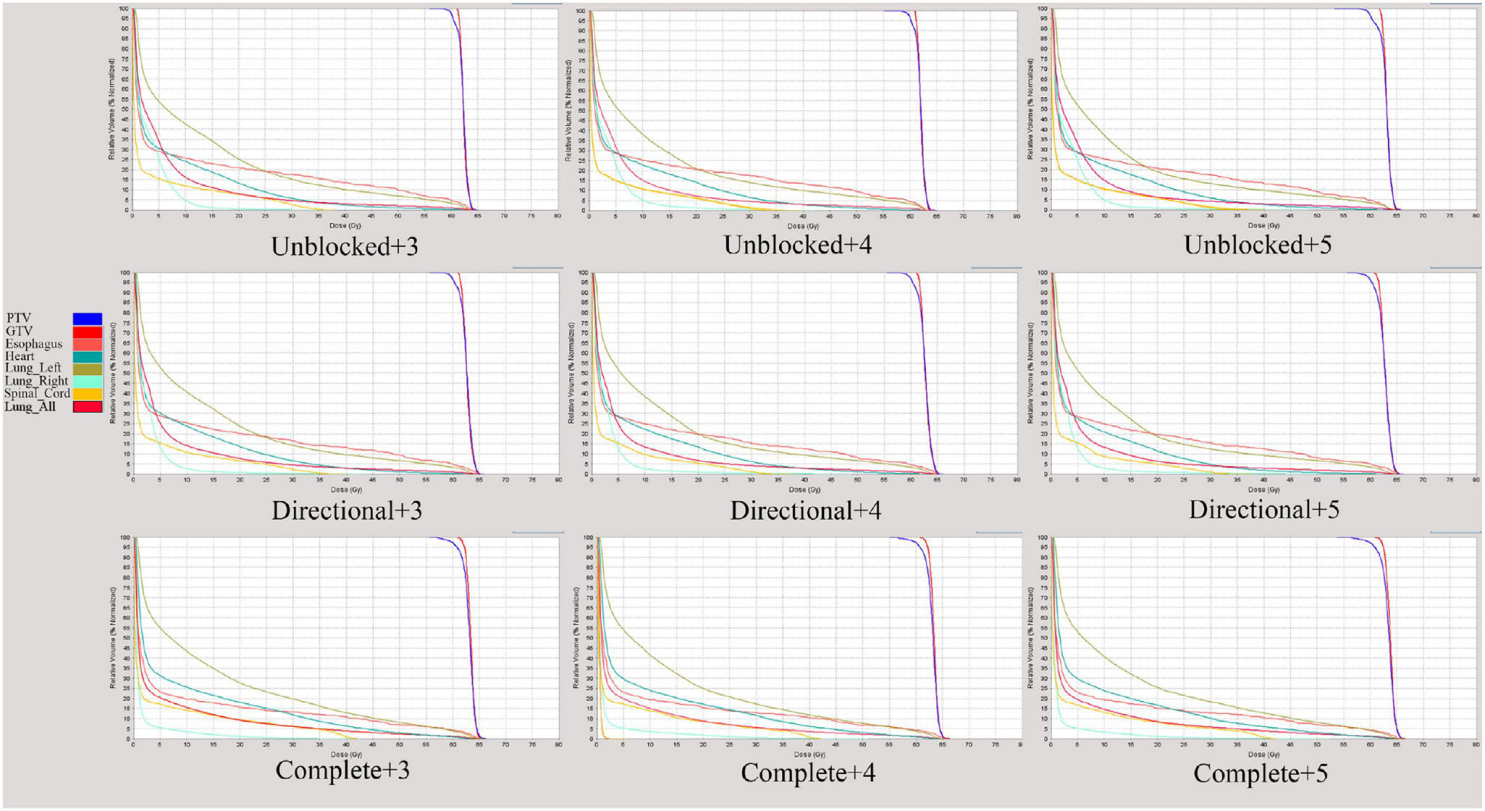
Various optimized combinations of dose–volume histograms in a patient. Note: Legend:Top‐right panel: Red contour = GTV; Blue contour = PTV Bottom‐left panel: Red = Bilateral lung, Brown = Spinal cord, Light green = Right lung, Yellow–green = Left lung, Dark green = Heart, Pink = esophagus.

Figures 2 and 3 show the dose distribution and DVH for the definitive treatment plan in a representative patient.Figures 2 and 3 demonstrate that progressively stricter block (Unblocked → Directional → Complete) significantly reduced the low‐dose bath V5 (light blue) and V10 (orange) for this patient. The reduction achieved by stricter block was more substantial than that achieved by increasing the MF.

### 3.2 Dose–volume parameters and treatment time for PTVs

Dosimetric parameters and treatment time of PTVs as indicated in Table 1. The average volume of both lungs is 68.21–137.93 cm^3^ (average 99.896 ± 20.709) cm^3^. The block and high MF have a small impact on the average dose of the target area. When applying a stricter block dose limit mode (Unblocked < Directional < Complete) and higher MF (3 < 4 < 5), HI increases (17.39%–27.54%), CI decreases (2.76%–17.43%), CI and HI have slightly worsened, but still meet the standard. Treatment time increased by 15.77%–131.58% (range: 3.21 ± 0.40 to 7.44 ± 1.28 min).

**TABLE 1:** Dosimetric parameters and treatment time for PTVs.

Block+MF	Dmean/Gy	HI	CI	Time (min)
Unblocked+3	62.365 ± 0.775	0.069 ± 0.020	0.832 ± 0.292	3.214 ± 0.398
Unblocked+4	62.354 ± 0.696	0.073 ± 0.019	0.820 ± 0.275	4.107 ± 0.501
Unblocked+5	62.297 ± 0.569	0.074 ± 0.015	0.809 ± 0.278	5.071 ± 0.581
Directional+3	62.356 ± 0.583	0.068 ± 0.018	0.829 ± 0.286	3.721 ± 0.485
Directional+4	62.731 ± 0.500	0.081 ± 0.013	0.808 ± 0.265	4.779 ± 0.630
Directional+5	62.766 ± 0.549	0.081 ± 0.017	0.788 ± 0.270	5.886 ± 0.766
Complete+3	62.911 ± 0.561	0.080 ± 0.013	0.739 ± 0.279	4.629 ± 0.771
Complete+4	63.032 ± 0.527	0.086 ± 0.012	0.706 ± 0.275	5.986 ± 1.039
Complete+5	63.146 ± 0.544	0.088 ± 0.011	0.687 ± 0.255	7.443 ± 1.284

### 3.3 Quantitative dose for lung

The dosimetric parameters for the bilateral lungs, healthy lungs (contralateral lung), and affected lung are shown in Tables 2, 3, and 4, respectively. Regarding V5, protection effectiveness: The block protection modes demonstrated a consistent hierarchy in reducing V5: Complete > Directional > Unblocked. Similarly, higher modulation factors offered greater protection: MF5 > MF4 > MF3. Organ‐specific impact: The block technique significantly reduced V5 in bilateral lungs and the healthy lung (*p* < 0.05), while its effect on the affected lung was minimal. Reductions from MF3 to MF4 were more substantial than from MF4 to MF5. The reduction from MF3 to MF4 is more pronounced than from MF4 to MF5. Combined effect: Combining block protection (especially Complete mode) with higher MF (MF4 or MF5) achieved the most effective V5 reduction. Mean reductions relative to the least protective settings (Unblocked + MF3) were: bilateral lungs 7.77%–33.13%, healthy lung 13.14%–69.38%, affected lung 3.18% (increase) to 3.20% (decrease).

**TABLE 2:** Quantitative dose indices for bilateral lung.

Block+MF	V5/%	Cohen's d	*p*	V10/%	Cohen's d	*p*	V20/%	Cohen's d	*p*	MLD/Gy	Cohen's d	*p*
Unblocked+3	36.235 ± 5.429			21.320 ± 2.987			12.522 ± 2.111			8.249 ± 0.946		
Unblocked+4	33.419 ± 5.225	1.803	^***^	19.732 ± 2.896	2.319	^***^	11.640 ± 2.004	2.415	^***^	7.904 ± 0.966	2.611	^***^
Unblocked+5	32.685 ± 4.423	1.651	^***^	19.032 ± 2.822	2.558	^***^	11.239 ± 2.061	2.557	^***^	7.766 ± 0.943	3.488	^***^
Directional+3	29.100 ± 5.259	1.453	^***^	19.782 ± 3.621	1.050	^***^	12.371 ± 2.265	0.358	–	7.841 ± 1.073	1.019	^***^
Directional+4	27.694 ± 4.930	1.930	^***^	18.377 ± 3.314	2.061	^***^	11.450 ± 2.136	2.014	^***^	7.551 ± 1.035	2.257	^***^
Directional+5	27.271 ± 4.939	1.977	^***^	17.959 ± 3.225	2.477	^***^	11.258 ± 2.074	2.233	^***^	7.485 ± 1.043	2.608	^***^
Complete+3	25.761 ± 5.221	2.076	^***^	19.469 ± 3.246	1.339	^***^	12.864 ± 2.195	0.373	–	7.625 ± 1.156	1.142	^***^
Complete+4	24.563 ± 4.761	2.359	^***^	18.081 ± 2.853	2.120	^***^	12.103 ± 2.023	0.403	–	7.357 ± 1.113	1.634	^***^
Complete+5	24.243 ± 4.681	2.457	^***^	17.814 ± 2.788	2.389	^***^	11.929 ± 2.005	0.618	^**^	7.317 ± 1.115	1.770	^***^

* *p <* *0.100*.

** *p <* *0.050*.

*** *p <* *0.010*.

‐ *p* > 0.100.

**TABLE 3:** Quantitative dose indices for healthy lung.

Block+MF	V5/%	Cohen's d	*p*	V10/%	Cohen's d	*p*	V20/%	Cohen's d	*p*	MLD/Gy	Cohen's d	*p*
Unblocked+3	26.398 ± 7.876			6.576 ± 3.224			1.446 ± 1.795			3.581 ± 0.870		
Unblocked+4	22.935 ± 7.221	1.279	^***^	5.371 ± 2.768	0.907	^***^	1.477 ± 1.752	0.095	^–^	3.388 ± 0.833	0.987	^***^
Unblocked+5	21.246 ± 5.468	1.328	^***^	4.525 ± 2.542	1.080	^***^	1.266 ± 1.642	0.411	^–^	3.232 ± 0.758	1.239	^***^
Directional+3	14.593 ± 6.536	1.513	^***^	4.446 ± 3.750	0.941	^***^	1.490 ± 2.175	0.064	–	2.921 ± 1.062	1.197	^***^
Directional+4	13.134 ± 5.686	1.851	^***^	3.613 ± 3.239	1.371	^***^	1.460 ± 1.997	0.023	–	2.816 ± 1.004	1.615	^***^
Directional+5	12.483 ± 5.520	1.926	^***^	3.359 ± 3.123	1.431	^***^	1.386 ± 1.914	0.088	^–^	2.763 ± 0.999	1.600	^***^
Complete+3	9.336 ± 6.344	1.957	^***^	4.451 ± 4.041	0.788	^**^	1.815 ± 2.568	0.380	^–^	2.016 ± 1.266	1.860	^***^
Complete+4	8.316 ± 5.590	2.120	^***^	3.454 ± 3.689	1.076	^***^	1.560 ± 2.383	0.110	^–^	1.851 ± 1.164	2.079	^***^
Complete+5	8.082 ± 5.523	2.180	^***^	3.323 ± 3.560	1.124	^***^	1.454 ± 2.367	0.008	^–^	1.834 ± 1.169	2.074	^***^

* *p* < 0.100.

** *p* < 0.050.

*** *p* < 0.010.

‐*p* > 0.100.

**TABLE 4:** Quantitative dose indices for affected lung.

Block+MF	V5/%	Cohen's d	*p*	V10/%	Cohen's d	*p*	V20/%	Cohen's d	*p*	MLD/Gy	Cohen's d	*p*
Unblocked+3	49.699 ± 4.152			41.558 ± 3.187			27.800 ± 2.164			14.676 ± 1.109		
Unblocked+4	48.226 ± 4.398	3.773	^***^	39.413 ± 3.633	2.437	^***^	25.594 ± 2.591	2.302	^***^	14.107 ± 1.286	2.146	^***^
Unblocked+5	47.997 ± 4.532	2.896	^***^	38.910 ± 3.828	2.013	^***^	24.964 ± 2.816	2.148	^***^	13.989 ± 1.348	2.006	^***^
Directional+3	49.367 ± 3.978	0.646	^***^	40.960 ± 2.897	0.827	^***^	27.296 ± 2.154	0.667	^*^	14.613 ± 1.076	0.217	^–^
Directional+4	47.788 ± 4.279	3.393	^***^	38.817 ± 3.216	2.964	^***^	25.099 ± 2.440	2.228	^***^	14.059 ± 1.196	1.66	^***^
Directional+5	47.406 ± 4.321	2.777	^***^	38.105 ± 3.430	2.702	^***^	24.740 ± 2.786	2.113	^***^	13.965 ± 1.290	1.567	^***^
Complete+3	48.911 ± 4.826	0.513	^*^	40.336 ± 3.901	0.792	^**^	28.170 ± 2.445	0.245	^–^	15.421 ± 1.230	1.217	^***^
Complete+4	47.396 ± 5.015	1.252	^***^	38.446 ± 3.931	1.827	^***^	26.741 ± 2.535	0.648	^**^	14.986 ± 1.315	0.437	^–^
Complete+5	47.162 ± 4.943	1.483	^***^	37.964 ± 4.118	1.972	^***^	26.393 ± 2.533	0.893	^***^	14.931 ± 1.344	0.37	^–^

* *p* < 0.100.

** *p* < 0.050.

*** *p* < 0.010.

‐*p* > 0.100.

Regarding V10, MF impact: Higher MF significantly reduced V10 (MF5 > MF4 > MF3), with the MF3‐to‐MF4 reduction being most pronounced. Block impact: Block protection reduced V10: Complete > Directional > Unblocked. Notably, the decrease in V10% from Directional to Unblocked is significant for both bilateral lungs and healthy lungs (*p* < 0.05), while there is no significant difference between Complete to Directional (*p* > 0.05).

Regarding V20, MF impact: V20 decreased significantly with higher MF (MF5 < MF4 < MF3), with larger reductions occurring between MF3 and MF4. Block impact: Block modes showed a complex effect: Directional block reduces V20, while Complete increases it, and Complete has a higher effect than Directional. Additionally, no significant difference in mean V20 was found for the healthy lung across techniques (*p* > 0.05).

Regarding mean lung dose (MLD), MF impact: MLD decreased significantly with higher MF (MF5 < MF4 < MF3), with the MF3‐to‐MF4 reduction being larger. Block impact: For bilateral and healthy lungs, MLD reduction followed: Complete < Directional < Unblocked. In the affected lung, Directional mode caused no significant MLD change versus Unblocked (*p* > 0.05), while Complete mode significantly increased MLD (*p* < 0.05).

**FIGURE 4:**
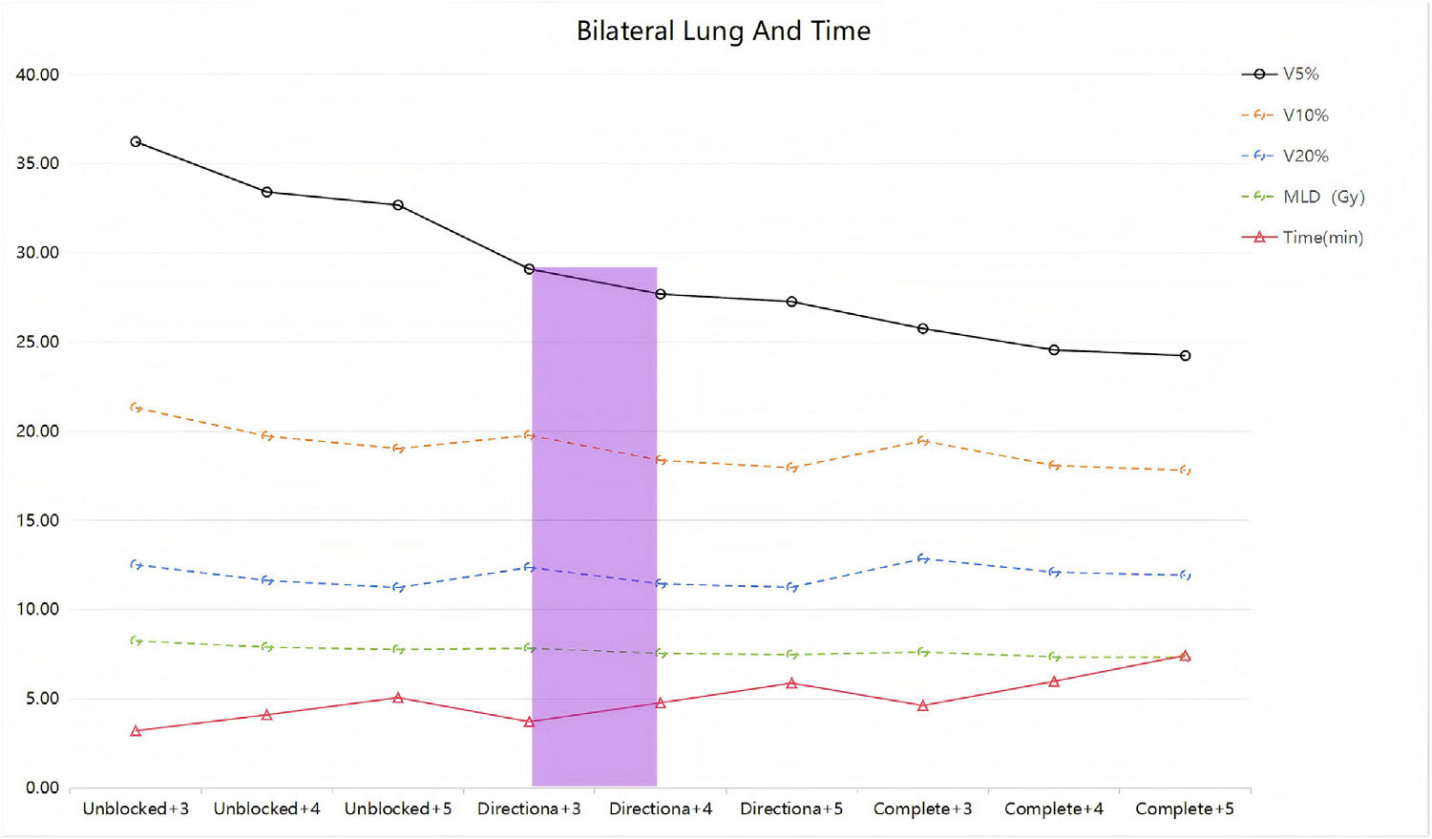
Comparison of the treatment time and quantitative dose indices for bilateral lung. Note:The lavender‐shaded region designates the clinically recommended configuration: Directional block with MF 3–4. From top to bottom: Solid black line: V5 (relative lung volume receiving ≥5 Gy); Amber dashed line: V10; Cobalt blue dashed line: V20; Viridian dashed line: MLD (mean lung dose); Cardinal red solid line: Treatment time(beam‐on time for a single 2 Gy fraction). MF, modulation factor; MLD, mean lung dose

A comparison of the treatment time and quantitative dose indices for bilateral lung (V5, V10, V20, MLD) is shown in Figure 4. Balanced approach: In nine treatment plans, the Directional  + MF3 and Directional  + MF4 plans offered a favorable balance: They significantly reduced low‐dose volumes (V5: 19.69%, 23.57%; V10: 7.21%, 13.80% reductions vs. Unblocked+MF3 baseline) while maintaining moderate increases in treatment time (15.77%, 48.69% increases). Target coverage: Plan quality metrics remained clinically acceptable: CI (MF3: 0.829 ± 0.286, *p* > 0.1; MF4: 0.808 ± 0.265, *p* > 0.05) and HI (MF3: 0.068 ± 0.018, *p* > 0.1; MF4: 0.081 ± 0.013, *p* < 0.01). While HI at MF4 showed a statistically increase, all values met institutional planning standards.

### 3.4 Quantitative dose indices for heart, esophagus and cord/max

The dosimetric parameters for the heart, esophagus, and cord/max are shown in Tables 5 and 6, respectively. The combined use of block and MF techniques had minimal impact on doses to the heart, esophagus, and cord (Dmax), with a slight increase in heart V30 was observed only for Complete+3 and 5 compared with Unblocked+3, rising from 4.00 to 5.42 and 5.23, respectively, and no statistically significant differences were found for the other combinations.

**TABLE 5:** Quantitative dose indices for heart.

Block+MF	V5/%	Cohen's d	*p*	V30/%	Cohen's d	*p*	V40/%	Cohen's d	*p*	MHD/Gy	Cohen's d	*p*
Unblocked+3	28.67 ± 16.24			4.00 ± 3.25			2.14 ± 1.78			6.16 ± 3.18		
Unblocked+4	29.26 ± 16.70	0.21	0.44	4.42 ± 3.30	0.55	0.06	1.97 ± 1.73	0.16	0.56	6.27 ± 3.20	0.24	0.39
Unblocked+5	26.33 ± 13.95	0.28	0.32	4.45 ± 3.16	0.54	0.06	2.07 ± 1.80	0.08	0.78	6.17 ± 3.11	0.01	0.98
Directional+3	28.57 ± 16.44	0.03	0.91	4.30 ± 2.54	0.20	0.46	1.87 ± 1.39	0.26	0.35	6.06 ± 2.84	0.14	0.60
Directional+4	28.16 ± 15.88	0.26	0.35	4.81 ± 3.09	0.57	0.05	2.23 ± 1.73	0.08	0.78	6.31 ± 3.12	0.25	0.38
Directional+5	28.00 ± 16.06	0.28	0.31	4.79 ± 3.35	0.50	0.08	2.46 ± 1.98	0.19	0.48	6.21 ± 3.20	0.07	0.79
Complete+3	27.17 ± 15.99	0.34	0.23	5.41 ± 4.13	0.79	0.01*	2.53 ± 2.23	0.25	0.37	6.41 ± 3.39	0.24	0.38
Complete+4	27.12 ± 16.05	0.30	0.29	8.50 ± 10.44	0.42	0.14	2.89 ± 2.34	0.48	0.10	6.48 ± 3.39	0.33	0.24
Complete+5	26.84 ± 15.82	0.32	0.26	5.23 ± 3.22	0.74	0.02*	2.64 ± 1.94	0.39	0.17	6.31 ± 3.14	0.15	0.60

p*** < 0.010, p** < 0.050, p* < 0.100, ‐*p* > 0.100.

**TABLE 6:** Quantitative dose indices for esophagus and cord/max.

Block+MF	V30/%	Cohen's d	*p*	V40/%	Cohen's d	*p*	V50/%	Cohen's d	*p*	Esophagus Dmean/Gy	Cohen's d	*p*	*Cord Dmax/Gy*	Cohen's d	*p*
Unblocked+3	16.06 ± 15.24			11.17 ± 14.02			9.16 ± 12.98			13.38 ± 7.65			31.89 ± 9.59		
Unblocked+4	18.08 ± 17.87	0.29	0.29	11.13 ± 13.80	0.09	0.73	8.91 ± 12.70	0.54	0.07	13.45 ± 8.16	0.08	0.79	32.50 ± 10.10	0.23	0.41
Unblocked+5	18.82 ± 19.07	0.31	0.26	12.72 ± 13.68	0.28	0.32	8.95 ± 12.84	0.43	0.13	13.56 ± 8.62	0.11	0.69	33.28 ± 9.30	0.41	0.15
Directional+3	17.56 ± 17.87	0.24	0.39	11.17 ± 14.27	0.00	0.99	9.02 ± 13.01	0.28	0.31	12.82 ± 8.62	0.42	0.14	31.48 ± 10.84	0.09	0.75
Directional+4	18.75 ± 20.06	0.26	0.34	14.15 ± 15.65	0.27	0.34	8.83 ± 12.90	0.39	0.16	13.29 ± 9.57	0.03	0.91	32.52 ± 10.65	0.11	0.68
Directional+5	18.67 ± 20.36	0.24	0.38	14.66 ± 16.82	0.26	0.35	10.18 ± 12.92	0.18	0.52	13.24 ± 10.03	0.04	0.89	33.30 ± 10.31	0.26	0.35
Complete+3	17.89 ± 20.45	0.17	0.54	14.33 ± 17.35	0.23	0.41	10.13 ± 13.36	0.16	0.55	12.08 ± 10.96	0.31	0.27	33.74 ± 9.96	0.35	0.21
Complete+4	18.33 ± 20.80	0.19	0.49	14.84 ± 18.32	0.23	0.41	11.74 ± 15.81	0.21	0.46	12.53 ± 11.84	0.15	0.59	35.36 ± 10.91	0.45	0.12
Complete+5	18.56 ± 20.87	0.22	0.43	15.08 ± 18.84	0.24	0.38	12.10 ± 16.62	0.22	0.42	12.81 ± 12.48	0.09	0.74	36.68 ± 10.72	0.56	0.06

p*** < 0.010, p** < 0.050, p* < 0.100, ‐*p* > 0.100.

## 4 DISCUSSION

Compared with IMRT, HT demonstrates superior conformity and homogeneity for the target volume in lung cancer treatment. However, this improvement in target coverage comes at the expense of increased volumes of low‐dose bath within the surrounding normal lung tissue. Retrospective analysis of 3986 patients with stage III lung cancer treated with radiotherapy through the SEER database found that the proportion of patients treated with IMRT increased year by year, with a significant decrease in lung V20; however, the incidence of adverse effects was similar, indicating that lowering V20 did not reduce the incidence of RP.^[^ ^15^ ^]^ Further studies found that V5 was the most effective predictor of symptomatic RP when treating lung lesions using HT techniques, with a V5 threshold of 65% for the development of symptomatic RP, and this study suggests that low doses of radiation to the lungs result in a higher risk of pulmonary toxicity reactions.^[^ ^8^ ^]^

Modern radiotherapy aims to precisely deliver a conformal dose with homogeneous target coverage while minimizing dose to OARs.^[^ ^16^ ^]^ HT, a specialized form of intensity‐modulated radiotherapy (IMRT), delivers radiation via a continuously rotating gantry combined with a binary pneumatic MLC. Unlike conventional linac‐based IMRT with static gantry angles, the dynamic delivery of HT achieves superior dose conformity and homogeneity, enabling enhanced precision in target coverage.^[^ ^17^, ^18^, ^19^, ^20^ ^]^ Although full‐arc delivery improves target conformity in deep‐seated tumors, it increases low‐dose spillage to peripheral normal tissues. Due to its low relative electron density (∼0.3), lung tissue exhibits increased lateral electron scatter and extended dose penumbra around solid tumors, amplifying low‐dose exposure.^[^ ^21^ ^]^ Consequently, mitigating low‐dose bath volumes in the lung—achieved through beam incidence modulation and optimization of delivery parameters (e.g., modulation factor)—is essential for patients undergoing HT.^[^ ^16^, ^22^ ^]^

To reduce the amount of unnecessary low doses received by normal lung tissue, the HT feature called block plan allows the use of virtual blocks to limit the beam during dose calculation. Zhu Haifu et al.^[^ ^23^ ^]^ showed that a half‐block in the healthy lung of a patient with unilateral lung cancer can effectively reduce the low‐dose bath in both lungs, but the arc should not be more than half the arc of the healthy lung. Chang et al.^[^ ^24^ ^]^ demonstrated that the application of a block model with a sector angle of 90°–140° in the HT program for esophageal cancer could be applied to most clinical cases, with larger sector angles resulting in greater dose reductions. Ito M et al.^[^ ^25^ ^]^ showed that semicircular blocks were more effective in reducing lung dose than fan‐shaped blocks and that the degree of dose reduction increased with increasing block size, with the most appropriate semicircular model showing good results in all patients in the study.

In addition, an appropriate MF can be set in the HT system to improve dose distribution and reduce the low‐dose bath volumes. The MF plays a crucial role by limiting leaf open times. MF represents the ratio of maximum to mean leaf open time for non‐zero projections. The MF parameter significantly impacts the duration of HT treatment and the quality of the treatment plan. However, a high MF value may lead to a longer beam‐on time.^[^ ^20^ ^]^ Increasing the MF improves dose distribution, however, beyond a threshold value, further MF elevation yields diminishing returns in OARs sparing while markedly prolonging beam‐on time.^[^ ^26^ ^]^ Shimizu et al. showed that the choice of MF depends on the difficulty of the case, recommending that planners use a high MF for difficult plans to improve modulation, such as cases involving complex target shapes around vulnerable organs. They retrospectively analyzed head–neck and prostate cancer cases to determine the upper limit of the MF for both tumors. By applying these thresholds, the mean MF values were optimized for both tumor types, significantly reducing treatment delivery time.^[^ ^27^ ^]^

In this study, we explore for the first time the combined application of block and optimized MF values to reduce the low‐dose bath of a spiral HT radiotherapy plan for lung cancer. A semi‐circular model with a block set to half the arc of the healthy lung showed a reduction in the low‐dose bath of the lung tissue in all patients, which is similar to the results of Ito et al.’s study in esophageal cancer. While the MF values were set at three values of 3, 4, and 5, the experimental results of this study were analyzed to determine the upper limit value interval of MF when applying the block. This was used as a reference to reduce unnecessarily high MF values, thus reducing treatment time and improving efficiency. Further studies found that higher MF had higher modulation capacity for better planning. Still, treatment time was significantly increased at MF as 4–5, while the percentage increase in modulation capacity was considerably lower. Further, we found that higher MFs have higher modulation capacity for better planning. Still, treatment time increased significantly with MF as 4–5, while the percentage increase in modulation capacity was substantially lower.

Combined application of semicircular block designed in the Directional block manner and MF in the 3–4 interval can effectively reduce the volume of V5 (19.69%, 23.57% reduction) and V10 in the low‐dose bath of both lungs (7.21%, 13.80% reduction), and have less effect on the HI and CI of the target area, with the HI at MF as 3 was 0.068 ± 0.018, CI was 0.829  0.286 (*p* > 0.1), treatment time was 3.721 ± 0.485, an increase of 15.77%, and at MF = 4, HI was 0.081 ± 0.013 (*p* < 0.01), CI was 0.808 ± 0.265 (*p* > 0.05), and the treatment time was 4.779 ± 0.630 min, 48.69%. Therefore, based on our findings, we recommend implementing Directional block (180° arc restriction) with a MF of 3–4 during helical tomotherapy planning for unilateral lung cancer. This protocol optimizes the trade‐off between OARs sparing and treatment efficiency.

Although the HT treatment plan recommended in this study for lung cancer patients with optimized block and MF parameters can effectively reduce the lung low‐dose bath, it has limitations: First, this study is still in the field of physical dosimetry and is based on the dosimetry study of radiation pneumonitis and has not been practically applied to the patients, and the combined application of block and MF to reduce the low‐dose bath in the spiral tomography of lung cancer. Therefore, whether the combined application of block and MF in HT radiotherapy to reduce the low‐dose bath can effectively reduce the probability of radiation pneumonitis in clinical practice needs to be further observed in conjunction with clinical practice before a more reliable conclusion can be drawn. Second, artificial intelligence has shown great potential in the current medical field, and has been studies on the automatic segmentation of the target area and organs at risk, the automatic generation of radiotherapy plans, and the prediction of radiotherapy plan dose in radiotherapy applications, and so on. If we collect enough excellent treatment plans for comparative analysis and establish a database, it will provide us with better and more accurate standards and templates for the setting of block and MF.

## 5 CONCLUSIONS

To balance target volume coverage (high dose), dose distribution uniformity, and treatment duration while reducing low‐dose irradiation to normal lung tissue, we recommend implementing directional block with a MF range of 3–4 to optimize the HT plan for patients with unilateral lung cancer.

## AUTHOR CONTRIBUTIONS

All authors contributed to the study conception and design. Material preparation, data collection, and analysis were performed by Sijin Zhu, Tianwen Zhang, Jiawen Yan, Yutao Zhao, Jinli Peng, Jingyan Gao, Yunyan Yang, Yan Xu, and Ya Li. The first draft of the manuscript was written by Tianwen Zhang and Jiawen Yan, and all authors commented on previous versions of the manuscript. All authors read and approved the final manuscript.

## CONFLICT OF INTEREST STATEMENT

The authors declare no conflicts of interest.

## ETHICS STATEMENT

Information of this clinical trial: This trial has been approved by the Ethics Committee of Yunnan Cancer Center. The file number is KYLX_2024‐042, registration date is February 07 2024, this is a retrospectively registration. This study was performed in accordance with the WMA ≪Declaration of Helsink≫ and CIOMS ≪International Ethical Guidelines for Biomedical Research Involving Human Subjects≫.

## Supporting information



Supporting Information

## Data Availability

The data that support the findings of this study are available from the authors but restrictions apply to the availability of these data, which were used under license from the Yunnan Cancer Center for the current study, and so are not publicly available. Data are, however, available from the authors upon reasonable request and with permission from the Yunnan Cancer Center.
