# Supplementary material for: Combined application of block and modulation factors to reduce the volume of the low dose area in helical tomotherapy plans for lung cancer
Source: J Appl Clin Med Phys. 2025 Dec 10;26(12):e70372. doi: 10.1002/acm2.70372 (PMC12695696; doi:10.1002/acm2.70372)
Supplement: Supplementary file 1 — Supporting Information [file ACM2-26-e70372-s001.docx]

| **Supplementary Table** Multivariate Test Results | | | | | | |
| --- | --- | --- | --- | --- | --- | --- |
|  | Testing Methodology | Proportion | *F* | *df1* | *df2* | *p* |
| Intercept | Pillai's Trace | 0.693 | 49.309 | 5 | 109 | 0.000 |
|  | Wilks' Lambda | 0.307 | 49.309 | 5 | 109 | 0.000 |
|  | Hotelling's Trace | 2.262 | 49.309 | 5 | 109 | 0.000 |
|  | Roy's Largest Root | 2.262 | 49.309 | 5 | 109 | 0.000 |
| Maximum Transverse Diameter of PTV | Pillai's Trace | 0.213 | 5.898 | 5 | 109 | 0.000 |
|  | Wilks' Lambda | 0.787 | 5.898 | 5 | 109 | 0.000 |
|  | Hotelling's Trace | 0.271 | 5.898 | 5 | 109 | 0.000 |
|  | Roy's Largest Root | 0.271 | 5.898 | 5 | 109 | 0.000 |
| Located | Pillai's Trace | 0.303 | 9.499 | 5 | 109 | 0.000 |
|  | Wilks' Lambda | 0.697 | 9.499 | 5 | 109 | 0.000 |
|  | Hotelling's Trace | 0.436 | 9.499 | 5 | 109 | 0.000 |
|  | Roy's Largest Root | 0.436 | 9.499 | 5 | 109 | 0.000 |
| Distance to Central Axis | Pillai's Trace | 0.582 | 30.340 | 5 | 109 | 0.000 |
|  | Wilks' Lambda | 0.418 | 30.340 | 5 | 109 | 0.000 |
|  | Hotelling's Trace | 1.392 | 30.340 | 5 | 109 | 0.000 |
|  | Roy's Largest Root | 1.392 | 30.340 | 5 | 109 | 0.000 |
| PTV Size/cm3 | Pillai's Trace | 0.353 | 11.885 | 5 | 109 | 0.000 |
|  | Wilks' Lambda | 0.647 | 11.885 | 5 | 109 | 0.000 |
|  | Hotelling's Trace | 0.545 | 11.885 | 5 | 109 | 0.000 |
|  | Roy's Largest Root | 0.545 | 11.885 | 5 | 109 | 0.000 |
| MF | Pillai's Trace | 0.807 | 14.889 | 10 | 220 | 0.000 |
|  | Wilks' Lambda | 0.205 | 26.393 | 10 | 218 | 0.000 |
|  | Hotelling's Trace | 3.829 | 41.356 | 10 | 216 | 0.000 |
|  | Roy's Largest Root | 3.814 | 83.910 | 5 | 110 | 0.000 |
| Block | Pillai's Trace | 0.976 | 20.948 | 10 | 220 | 0.000 |
|  | Wilks' Lambda | 0.157 | 33.217 | 10 | 218 | 0.000 |
|  | Hotelling's Trace | 4.525 | 48.871 | 10 | 216 | 0.000 |
|  | Roy's Largest Root | 4.330 | 95.265 | 5 | 110 | 0.000 |
| MF:Block | Pillai's Trace | 0.087 | 0.498 | 20 | 448 | 0.967 |
|  | Wilks' Lambda | 0.914 | 0.497 | 20 | 362 | 0.967 |
|  | Hotelling's Trace | 0.093 | 0.498 | 20 | 430 | 0.967 |
|  | Roy's Largest Root | 0.077 | 1.730 | 5 | 112 | 0.134 |
| NOTE：Wilks' Lambda Proportion = Sum of Squares Within (SSW) / Total Sum of Squares (SST) | | | | | | |
